# Initial Dietary Protein Intake Influence Muscle Function Adaptations in Older Men and Women Following High-Intensity Interval Training Combined with Citrulline

**DOI:** 10.3390/nu11071685

**Published:** 2019-07-22

**Authors:** Fanny Buckinx, Vincent Marcangeli, Lívia Pinheiro Carvalho, Maude Dulac, Guy Hajj Boutros, Gilles Gouspillou, Pierrette Gaudreau, José Morais, Philippe Noirez, Mylène Aubertin-Leheudre

**Affiliations:** 1 Department of Exercise Science, Groupe de Recherche en Activité Physique Adapté (GRAPA), Université du Québec à Montréal, Montréal, QC H2X 1Y4, Canada; 2 Centre de Recherche de l’Institut Universitaire de Gériatrie de Montréal, Montréal, QC H3W 1W6, Canada; 3 WHO Collaborating Centre for Public Health Aspects of Musculoskeletal Health and Ageing, 4000 Liège, Belgium; 4 Department of biology, Université du Québec à Montréal, Montréal, QC H2X 1Y4, Canada; 5 Centre de Recherche du Centre Hospitalier Universitaire de Montréal and Département de médecine, Université de Montréal, Montreal, QC H3T 1J4, Canada; 6 The Research Institute of the McGill University Health Centre and Division of Geriatric Medicine, McGill University, Montreal, QC H3A0G4, Canada; 7 Université Paris Descartes, Institut de Recherche bioMédicale et d’Épidémiologie du Sport (IRMES), 75015 Paris, France

**Keywords:** HIIT, citrulline, protein intake, functional capacities, muscle function, aging

## Abstract

Background: This study evaluates whether the initial amount of dietary protein intake could influence the combined effect of high-intensity interval training (HIIT) and citrulline (CIT), or HIIT alone, on body composition, muscle strength, and functional capacities in obese older adults. Methods: Seventy-three sedentary obese older men and women who completed a 12-week elliptical HIIT program with double-blinded randomized supplementation of CIT or placebo (PLA) were divided into four groups according to their initial protein intake (CIT–PROT+: *n* = 21; CIT–PROT−: *n* = 19; PLA–PROT+: *n* = 19; PLA–PROT−: *n* = 14). Body composition (fat and fat-free masses), handgrip (HSr) strength, knee extensor (KESr) strength, muscle power, and functional capacities were measured pre-intervention and post-intervention. Results: Following the intervention, the four groups improved significantly regarding all the parameters measured. For the same initial amount of protein intake, the CIT–PROT− group decreased more gynoid fat mass (*p* = 0.04) than the PLA–PROT− group. The CIT–PROT+ group increased more KESr (*p* = 0.04) than the PLA–PROT+ group. In addition, the CIT–PROT− group decreased more gynoid FM (*p* = 0.02) and improved more leg FFM (*p* = 0.02) and HSr (*p* = 0.02) than the CIT–PROT+ group. Conclusion: HIIT combined with CIT induced greater positive changes than in the PLA groups. The combination seems more beneficial in participants consuming less than 1 g/kg/d of protein, since greater improvements on body composition and muscle strength were observed.

## 1. Introduction

Normal aging is known to derive from an accumulation of detrimental changes such as the loss of muscle mass, strength, and quality, leading to a poor physical function [1,2,3]. Exercise is one of the most promising non-pharmacological strategies to counteract physical declines [4]. Exercise intervention has been recently recommended by the World Health Organization (WHO) to be the best strategy to reverse these declines, leading to healthy aging [5]. However, almost 60% of older adults are sedentary [6], which is mainly due to a perceived lack of time to practice physical activity [7]. Considering this barrier, high-intensity interval training (HIIT), a short but intense physical activity training recognized to improve metabolic functions, aerobic capacity, and body composition in adults [8,9,10], could be considered as a beneficial strategy to counteract these aging processes in obese older adults.

Another potential non-pharmacological strategy to manage the loss of physical function among older adults is to consider dietary interventions. L-citrulline (CIT; a non-essential alpha-amino acid found in high levels in watermelon [11]), has recently raised the interest as a potential beneficial in older adults [12,13], since its supplementation leads to positive effects on muscle function [14,15,16] and fat metabolism [17] in older rats.

Previous investigations suggest a beneficial additive effect of CIT supplementation when combined with exercise training on muscle function in obese older adults. In this sense, Figueroa et al. showed that citrulline (CIT) supplementation combined with physical exercise on a vibration platform increased more significantly leg fat-free mass in obese post-menopausal women compared to the control group (i.e., L-citrulline alone) [18]. Very recently, our team also highlighted that CIT supplementation combined with HIIT increases further muscle strength and gait speed than HIIT alone, in dynapenic-obese older men and women [19].

It has also been reported that CIT supplementation could be efficiently improve muscle function in healthy adults, depending on the initial amount of dietary protein daily intake [20]. In this pilot study, the oral administration of CIT stimulated muscle protein synthesis in participants consuming a low amount of dietary proteins (i.e., protein 8% (or 0.69 g/kg/day), carbohydrates 60%, and fat 32% by calories) [20]. Bouillane et al. also highlighted that CIT supplementation was associated with a higher systemic amino acid availability in malnourished older adults [13]. To our knowledge, few studies have examined the effects of CIT in healthy or obese older adults. Moreover, evidence suggests that the efficacy of an exercise intervention on muscle function could also be influenced by the initial amount of protein intake. For example, Dulac et al. proposed that healthy older men should ingest at least 1.2 g/kg/day of protein in order to optimize the effect of a mixed power training on muscle function and quality (i.e., a predictive factor of functional capacities in older adults) [21]. Bradlee et al. also concluded that a higher animal protein intake combined or not with a healthy active lifestyle (i.e., including the time spent per day to sleep, in sitting position, in slight/moderate/heavy activity), is associated with a better maintenance of muscle mass and functional performance in older men and women [22]. According to Buckinx et al., 2019, if obese older adults are following recommended dietary protein allowances, eating 20 g of proteins in each meal does not further improve muscle adaptation in response to the HIIT intervention [23]. Altogether, the effects of protein intake on muscle adaptation following an exercise intervention remain unclear.

Therefore, the aim of the present study was to evaluate, a posteriori, if the initial amount of daily protein intake could influence the combined effect of HIIT and CIT on functional capacities, physical endurance, muscle strength, power, and body composition in obese older men and women.

## 2. Methods

### 2.1. Study Design and Population

To address this question, we used an a posteriori design, based on a double-bind randomized controlled trial (RCT NCT02417428), comparing the effects of HIIT alone versus HIIT combined with L-citrulline supplementation on functional capacity and muscle function in obese elderly men and women [19].

Recruitment of participants took place in Montreal (Canada), in the community via social communication (flyers and meetings in community centers). The inclusion criteria of the study “RCT NCT02417428” were considered for the present analysis: (1) to be aged 60 years and over, (2) inactive during the last 6 months (<2 h/week of structured exercise), (3) obese (fat mass [FM]: men >25%, women >35% [24]), (4) to have a stable weight (±2 kg) over the past 6 months, (5) no orthopedic limitations, (6) no counter-indication to practice physical activity (Physical Activity Readiness Questionnaire), (7) no menstruation for the past 12 months for women, (8) non-smoker and (9) non-alcohol consumers (≥2 drinks/day). Participants with diagnosed neurological, cardiovascular, lung diseases, or cognitive disorders were also excluded.

All the participants who completed a 12-week elliptical high-intensity interval training (HIIT) program and take a double-blinded randomized supplementation (CIT or placebo (PLA)) were a posteriori divided into four groups according to the initial amount of protein intake (PROT-: <1 g/kg/day or PROT+: >1 g/kg/day): (1) CIT–PROT+: *n* = 21; (2) CIT–PROT-: *n* = 19; (3) PLA–PROT+: *n* = 19 and (4) PLA–PROT−: *n* = 14. The cut-off value of 1 g/kg/day was chosen based on the study of Granic et al. suggesting that intake of <1 g/kg/day may negatively affect muscle strength and physical performance in late life [25].

The protocol was approved by the Ethics Committee of the “Université du Québec à Montréal” (UQAM). All the participants gave informed consent to be enrolled in this study.

### 2.2. Intervention

The intervention consisted of high-intensity interval training (HIIT) combined with citrulline (CIT) or placebo (PLA).

The HIIT consisting of 12 weeks of training supervised by a kinesiologist. Three session of 30 min were performed each week (on non-consecutive days) on an elliptical device to reduce, in this obese population, lower extremity joint impact [26]. The percentage of maximal heart rate (MHR) and/or the perceived exertion (Borg scale) were used to estimate the intensity of the session [27]. The MHR was determined using the validated equation of Karvonen [((220 − age) − HR rest) × % HR target] + HR rest [28]. The training sessions (30 min) included: (1) 5 min of warm-up at a low intensity (50%–60% MHR and/or a 6 score between 8–12 on the Borg scale); (2) 20 min of HIIT consisting of multiples 30-s sprints at a high intensity (80–85% MHR or Borg scale score > 17) alternating with sprints of 90 sec at a moderate intensity (65% MHR or Borg scale score of 13–16); and (3) 5 min of cool-down (50%–60% MHR and/or a Borg scale score 8–12). Note that adjustments of speed and resistance of the elliptical device could be made at any time during the session so that MHR was always above 80% during high-intensity intervals. Participants who did not complete at least 80% of the training sessions were excluded from the final analysis [26].

During the 12-week intervention, participants took also every day during the lunch and blindly a single daily dose of 10 g of L-CIT (Citrage©, Créteil, France) containing 38 kcal per dose or a single dose of 10 g of placebo powder (maltodextrin) equivalent in weight, appearance, taste, and calories. The dose of CIT ingestion was based on the results of Moinard et al. [29].

### 2.3. Evaluations

Assessment were performed at the «*Département des Sciences de l’activité physique*, *Université du Québec à Montréal (UQAM)*». Functional capacities, physical endurance, muscle strength, muscle power, body composition, and lifestyle habits (dietary intake and physical activity level) were evaluated pre-intervention and post-intervention.

#### 2.3.1. Dietary Intake

The three-day (two weekdays and one weekend day) food record method was used to assess dietary intake [30]. Participants were asked to keep their dietary habits during the intervention period, the same. Based on the standardized Canadian Food file (CNF2015 [31]), total intakes, protein, lipids, and carbohydrate were calculated using the software Nutrific© (Université Laval, Québec, QC, Canada). Participants were divided a posteriori in four groups according to the initial amount of protein intake (PROT−: <1 g/kg/day versus PROT+: >1 g/kg/day) [25]: (1) CIT–PROT+: *n* = 21; (2) CIT–PROT−: *n* = 19; (3) PLA–PROT+: *n* = 19 and (4) PLA–PROT−: *n* = 14.

#### 2.3.2. Functional Capacities

**Timed up and go test:** To assess walking speed, the «Timed Up and Go» test (in seconds) was used at a comfortable and self-paced (TUG) and at a fast-paced walking speed (TUGf). The participants were instructed to sand from a chair, walk a 3-meter distance, and sit down again [32], A duration above 30 s indicates limited mobility and an increased risk of falling, whereas a duration of less than 20 s indicates appropriate mobility, with the subject likely to be independent in activities of daily living [33].

**Chair stand test:** To assess lower-body function, the chair stand test was performed. This test consists of standing up and sitting down with arms folded across their chest. The time (in seconds) required to complete the task 10 times was recorded [34].

**Alternate-step test:** This test was used to estimate the ability of weight shifting in the forward and upward directions. To do this, participants were asked to touch the top of a 20-cm height step with separate feet alternately, as fast as possible during a 20-s period [35,36]. The number of step counts was recorded for analysis.

**Unipodal balance test:** To assess balance, the validated unipodal balance test was used. This test consists of standing alternately on one foot (right and left), with eyes opened and arms along the body. The time (in seconds) was recorded from the moment one foot was lifted from the floor to the moment when it touched the ground, the stance leg moved, or until 60 s had elapsed [37].

**Physical Endurance:** To assess mobility and aerobic capacities, the 6-minute walking test (6MWT) was used. Participants were instructed to walk the longest distance possible during 6 min. Based on the American Thoracic Society (ATS)/American College of Chest Physicians recommendations for the six-minute walking test, participants received the same standardized encouragement, every minute of the test [32]. During the test, participants had the possibility of interrupting and return to exercising and slowing down or speeding up, according to perceived effort [38]. The covered distance (in meters) during the test served as an indicator of mobility capacity. In addition, the VO2 max (in mL/kg/min) was estimated and served as an indicator of aerobic capacity. To estimate the VO2, the following validated equation was used: 70.161 + (0.023 × distance [m]) − (0.276 × body weight [kg]) − (6.79 × sex [Men = 0, Women = 1]) − (0.193 × HR [pulse/min]) − (0.191 × age [years]) [39].

#### 2.3.3. Muscle Strength and Power

**Handgrip strength:** To assess the maximal isometric upper limb muscle strength, handgrip strength (HS) was used by means of a handheld dynamometer with adjustable grip (Lafayette© Instrument, Sagamore Pkwy N, IN, USA) [40]. In a standing position with arms along the body, participants were asked to squeeze the dynamometer as hard as possible during 4 s. Three measurements for each hand, alternately, were performed, and the best score of each was recorded for the analysis. The relative value (HSr: divided by body weight (BW; kg/kg)) of upper limb muscle strength was reported, since it is more related to functional incapacities and avoids sex effects [41].

**Knee extensor strength (KES):** To assess the maximal isometric lower limb muscle strength, knee extensor strength was used by means of a strain gauge system attached to a chair (Primus RS Chair, BTE) upon which participants were seated with the hip joint angle set at 90°. The knee angle was increase to 135° in order to lower the maximal joint torque [42,43], specifically in the elderly population who presented low bone density and a high risk of fracture [44]. The tested leg was fixed to the lever arm at the level of the lateral malleoli on an analog strain gauge to measure strength. The highest of three maximum voluntary contractions was measured [45]. Lower limb muscle strength was also expressed relative to body weight value (KESr: N/kg).

**Muscle Power:** Using the Nottingham Leg Extensor Power rig, lower limb muscle power was recorded in a sitting position [46]. As previously described and validated, participants were asked to push the pedal down as hard and fast as possible until a plateau/decrease was observed [47]. The better push was used to express muscle power (W).

#### 2.3.4. Body Composition

**Anthropometric measures:** Body mass index (BMI = Body mass (Kg)/Height^2^ (m^2^)) was determined by measuring body weight and height using an electronic scale (Omron HBF-500CAN) and a stadiometer (Seca). Waist circumference (WC) was measured at the level of the umbilicus (belly button) to the nearest 0.1 cm.

**Body composition (DXA):** Total fat mass (FM (%)), android fat mass (i.e., distribution of human adipose tissue mainly around the trunk and upper body, in areas such as the abdomen, chest, shoulder, and nape of the neck, (%)), gynoid fat mass (fat around the hips, thighs, and bottom (%)), total lean body mass (LNM (kg)), leg lean mass (LLM (kg)), and appendicular lean mass (App LM (kg/m²)) were quantified by dual-energy X-ray absorptiometry [DXA] using a Lunar Prodigy whole-body scanner (GE Medical Systems, Madison, WI, USA). Age, height, and weight allow to estimate the thickness of the subject to automatically alter scan depth. The participants were wearing light indoor clothing and no removable metal objects during all scans. The scan time was about 5 min, depending on the height.

**Muscle composition (pQCT):** Muscle composition was assessed using a peripheral quantitative computed tomography scan (pQCT; Stratec XCT3000 STRATEC Medizintechnik GmbH, Pforzheim, Germany, Division of Orthometrix; White Plains, NY, USA) of the right leg and was obtained using the 66% distance of the femur (starting from the lateral trochanter down to the lateralepicondyle). The total length, voxel size (0.5 mm), and speed (10 mm/s) were entered as scanning parameters. Following Bone Diagnostics, Inc. (Fort Atkinson, WI, USA) guidelines, all the pQCT scans were acquired by trained operators. After data acquisition, a second evaluator visually assessed image quality to allow for analysis. The visual inspection rating scale classified all the images as a rate up to three, according to a previously reported visual scale of movement artefact [48]. The open source software ImageJ (version 1.51q) with the plugin BoneJ (Version 1.3.11) was used [49]. To lower noise, BoneJ’s soft tissue analysis uses a median filter of 7 × 7. The tissue thresholds selection allows discriminating soft tissue as well as bone area and density. Muscular, bone, intramuscular adipose tissue (IMAT), and subcutaneous adipose tissue thresholds were defined based on the parameters of a previous study [50]. Thus, four data were collected: lean muscle area (cm²), total fat area (cm²), subcutaneous fat area (cm²), and intramuscular fat area (cm²).

### 2.4. Statistical Analysis

The Kolmogorov test was used to test data distributions. Qualitative variables were expressed as percentage and quantitative variables were expressed as mean ± standard deviation (SD). One-way analysis of variance (ANOVA) followed by Bonferroni’s post-hoc comparisons tests were used to compare the four groups (CIT–PROT− versus PLA–PROT− versus CIT–PROT+ versus PLA–PROT+) at baseline. Then, a GLM repeated measures (2*2) and post hoc Bonferrroni analysis were performed to determine the effects of the intervention. Finally, the evolution of the parameters according to the initial amount of protein and according to the supplementation (delta change = post–pre*100) were calculated and compared. Intra-group (paired *t*-test) and inter-group (Mann–Whitney test) evaluations were also performed to compare groups according to the amount of protein and supplementation in order to avoid a potential lack of power. All the statistical analyses were performed using SPSS 25.0 (IBM, Chicago, IL, USA). *p* < 0.05 was considered statistically significant.

## 3. Results

### 3.1. Population

Out of the 107 participants eligible for the initial double-blind randomized controlled trial [19], 83 completed the 12-week HIIT intervention (i.e., nine subjects refused to participate, and 15 subjects were excluded (based on our selection criteria) because the adherence to training sessions was <80%).

**Figure 1:**
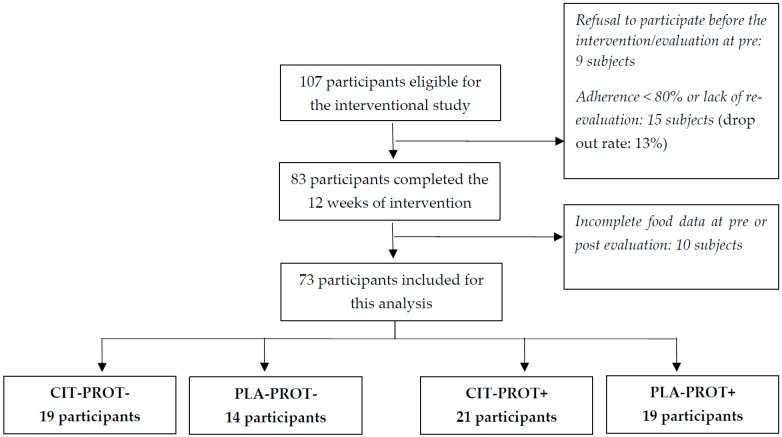
Flow chart of the study.

Ten of them have been excluded from this analysis because of incomplete food data. Therefore, 73 sedentary obese participants were, a posteriori, divided into four groups, according to their initial amount of protein intake: PROT−: <1 g/kg/day versus PROT+: 1 g/kg/day, and to the randomized supplementation group: CIT versus PLA (Figure 1).

Baseline characteristics of the 73 participants, according to the four groups, are shown in Table 1. Groups were comparable for most variables. However, the body weight in the PLA–PROT− group was significantly higher than the CIT–PROT+ and PLA–PROT+ groups (*p* = 0.038 and *p* = 0.013, respectively). The BMI was also significantly higher in the PLA–PROT− compared to the PLA–PROT+ group (*p* = 0.03). As expected by the design of the study, the CIT–PROT− and PLA–PROT− groups consumed significantly less protein than the CIT–PROT+ and PLA–PROT+ groups (*p* < 0.001). Finally, CIT–PROT+ group had higher energy intake compared to the CIT–PROT− (*p* = 0.015) and PLA–PROT− (*p* = 0.003) groups. Therefore, all the following statistical analysis were adjusted on BMI. Note that no other baseline difference between groups was observed for all the measured variables (i.e., body composition, strength, and functionality).

### 3.2. Effects of Initial Protein Intake

The influence of initial amount of protein intake on the combined effect of HIIT and CIT regarding functional capacities, physical endurance, muscle strength, muscle power, and body composition in a population of obese older adults is shown below.

### 3.3. Functional Capacities and Physical Endurance

Globally, HIIT improved the functional capacities (*p* < 0.001 for all parameters). Moreover, when the effect is analyzed intra-group via paired t-tests, we observe that all the groups significantly improved their functional capacities. Nevertheless, no difference between Time*group(s) was observed (Table 2). The intervention (HIIT) also significantly improved the physical endurance evaluate using the 6MWT (*p* < 0.001). The four groups increased significantly their distance covered in 6 min. However, no group*time effect was highlighted (*p* = 0.70; Table 2).

### 3.4. Muscle Strength and Power

As shown in Table 3, HIIT significantly improved muscle strength (p time effect). The grip strength also improved between groups (*p* = 0.04; time*group effect). However, the post hoc analyses could not identify a significant difference between specific groups. In addition, we observe a trend of improvement for lower limb muscle strength between groups (*p* = 0.07). A possible explanation for the absence of significance is a lack of power (<0.05). Furthermore, only the CIT–PROT− group significantly improved the relative handgrip strength (0.41 versus 0.45 kg/kg, *p* < 0.001) and only the CIT–PROT+ group significantly improved the relative knee extensor strength (9.9 versus 11.4 N/kg, *p* = 0.001). Finally, HIIT improved significantly lower limb muscle power (*p* < 0.001). However, no effect between groups was observed (*p* = 0.91; Table 3).

### 3.5. Body Composition

Following the intervention (time effect), body composition variables were significantly improved. Nevertheless, no group effect was observed for these variables. However, we note that an inter-group difference trend with regard to gynoid fat mass is observed (*p* = 0.06). When the effect is analyzed intra-group via paired t-tests, we observe that total fat (38.4% versus 37.1%, *p* = 0.004) and gynoid fat (41.2% versus 39.0%, *p* = 0.002) percentages significantly decreased in the CIT–PROT− group. In this group, leg lean mass also significantly increased (16.4 kg versus 16.9 kg, *p* = 0.019). A significant reduction in total fat mass was also highlighted in CIT–PROT+ group (38.4% versus 37.1%, *p* = 0.004) whereas no significant difference in body composition for group PLA− PROT+ was observed (Table 4).

### 3.6. Evolution of the Parameters According to the CIT Supplementation

A comparison between supplementation groups (CIT versus PLA) with the same initial protein intake (prot + or prot −) was also performed (Table 5).

**Figure 2:**
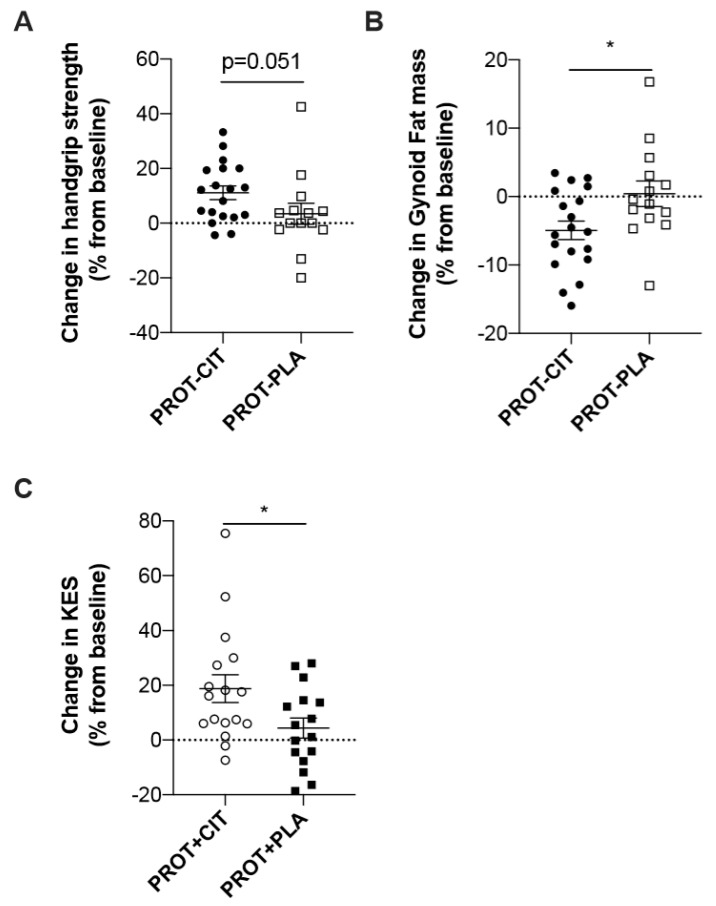
Evolution of the parameters according to the supplementation. (**A**) Percentage change of handgrip strength, according to the supplementation (PLA vs. CIT) in low-protein intake groups (*p* = 0.02). (**B**) Percentage change of gynoid fat mass, according to the supplementation (PLA vs. CIT) in low-protein intake groups (*p* = 0.04). (**C**) Percentage change of knee extensor isometric strength (KES), according to the supplementation (PLA vs. CIT) in high-protein intake groups (*p* = 0.04). * Difference statistically significant (*p* < 0.05).

We observe a trend toward improving relative grip strength in the CIT–PROT− group compared to the PLA–PROT− group (*p* = 0.051, Figure 2a). This difference was clinically significant (≥5%). Moreover, the decrease in gynoid fat mass was significantly greater in the CIT–PROT− group than in the PLA–PROT− group (*p* = 0.04, Figure 2b). Finally, a higher improvement in knee extensors strength was observed in the CIT–PROT+ group compared to the PLA–PROT+ group (*p* = 0.04, Figure 2c).

### 3.7. Evolution of the Parameters According to the Initial Amount of Dietary Protein Intake

A comparison between groups with the same supplementation (i.e., CIT or PLA) but with a different initial amount of protein intake (prot + versus prot –) was also performed (Table 6).

**Figure 3:**
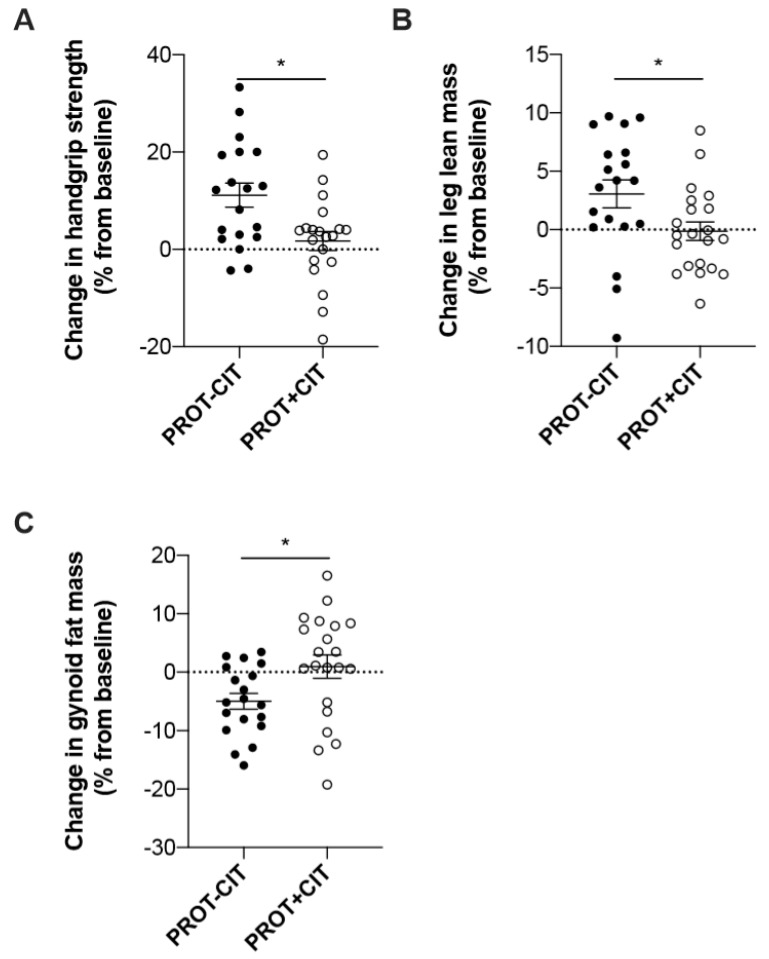
Evolution of the parameters according to the initial amount of protein intake. (**A**) Percentage change of handgrip strength, according to the initial amount of protein intake. (**B**) Percentage change of leg lean mass, according to the initial amount of protein intake. (**C**) Percentage change of gynoid fat mass, according to the initial amount of protein intake. * Difference statistically significant (*p* < 0.05).

The CIT–PROT− group improved significantly more of its relative grip strength (*p* = 0.02, Figure 3a) and its leg lean mass (*p* = 0.02, Figure 3b) than the CIT–PROT+ group. The higher improvement of relative grip strength observed in the CIT–PROT− is statistically but also clinically (≥5%) significant. Finally, the CIT–PROT− group decreased significantly more of its gynoid fat mass compared to the CIT–PROT+ group (*p* = 0.02, Figure 3c).

However, in the placebo groups, no significant difference between the groups was observed according to the initial amount of protein intake.

## 4. Discussion

Despite a small sample size, the present study highlights, for the first time, that the initial amount of protein intake could influence the combined effect of CIT and HIIT in a population of obese older adults. First, for the same initial amount of protein intake, CIT–PROT− decreased more gynoid FM (*p* = 0.04), tended to decrease more total fat area (*p* = 0.06), and tended to increase more HSr (*p* = 0.051) than the PLA–PROT− group, whereas the CIT–PROT+ group increased more KESr (*p* = 0.04)) than the PLA-PROT+ group. More interestingly, the results suggest that the combination of HIIT+CIT is more beneficial in obese older adults eating initially less than 1 g/kg/day of proteins daily, since they had greater improvements on body composition and handgrip strength. Indeed, CIT–PROT− decreased significantly more gynoid FM (CIT–PROT−: −4.9% versus CIT–PROT+: 0.9%, *p* = 0.02), improved significantly more leg FFM (CIT–PROT−: 3.1% versus CIT–PROT+: −0.1%, *p* = 0.02) and HS (CIT–PROT−: 12.4% versus CIT–PROT+: 3.0% g) and tend to improve significantly more appendicular lean mass (CIT–PROT−: 1.24 % versus CIT–PROT+: 0.88%, *p* = 0.05) than CIT–PROT+. In placebo groups, the initial protein intake did not seem to have any impact on others’ physical and functional parameters. In summary, our results suggest that the combination of HIIT and CIT induces a greater anti-obesity effect than HIIT alone, in a population of older obese adults, while maintaining muscle function. This is in line with the literature highlighting the importance of making efforts to promote healthy aging by consider both preventing obesity and maintaining or increasing skeletal muscle mass and function [51].

It has recently been reported that resistance training results in higher muscle protein synthesis than endurance training in older healthy men [52]. In this sense, Dulac et al. indicated that higher protein consumption leads to better muscle adaptation following mixed power training [21]. The absence of positive results on a number of physical and functional variables in the present study is inconsistent with those of Dulac et al. and could be explained by several hypotheses [21]. First, the training mode is different between the studies (i.e., HIIT versus mixed power training). Based on these results, we can assume that protein intake may play a less important role in muscle adaptations following HIIT than other types of physical training, since it is less effective in protein synthesis and thus in the improvement of muscle function in older people. In addition, in a randomized controlled trial, Mitchell et al. also concluded that men ingesting a higher quantity of protein (i.e., 1.6 g/kg/day) during 10 weeks displayed a significantly greater improvement in their total muscle mass (i.e., +1.49 kg versus 0.55 kg, *p* < 0.001) and muscle power (i.e., +26.6 W versus −11.7 W, *p* = 0.015) than those having less protein intake (i.e., 0.8 g/kg/day) [53]. So, Mitchell et al. as Dulac et al. suggested that initial protein intake has a modulatory role in improving or maintaining muscle function. Nevertheless, Moro et al. have shown that healthy older adults do not seem to be affected by anabolic resistance [54]. In this sense, a lack of difference between groups could be explained by the participant’s health variables, since our population has less health detrimental. A second hypothesis that could explain the differences between our results and those of the previous studies is the sex, since they only have men, whereas we included both sexes [21,53]. A recent study reported that muscle quality (8.8% for women versus 33.8% men, *p* < 0.05) and maximal quadriceps strength (15.8% for women versus 41.7% for men, *p* < 0.05) adaptations following an 18-week resistance training are two times less important in women than in men [55]. Based on this research, the discrepancies between our study and the studies of Dulac et al. and Mitchell et al. could be influenced by our sample including men and women equally [21,53]. Indeed, the lack of significance in our results could be due to the proportion of women in our study (~50%). Please note that a sex-based analysis could not be performed because of the number of subjects (n too small to be statistically relevant).

However, it is important to note that we observed significant differences in relative grip strength and gynoid fat mass between the CIT–PROT− and PLA–PROT− groups, as well as between the CIT–PROT− and CIT–PROT+. These differences (i.e., CIT–PROT− versus CIT–PROT+) may be the result of an interaction between the amount of protein consumed and the CIT rather than an interaction between the amount of protein and exercise adaptations. Indeed, our results on body composition parameters and muscle function (i.e., grip strength) are in line with the studies of Jourdan et al. and Osowska et al. showing that muscle protein synthesis was significantly improved in individuals supplemented with CIT and initially consuming a low quantity of protein, compared to individuals supplemented with non-essential amino acids [14,20]. In addition, a recent study shows that even if there is no difference in muscle protein synthesis, a three-week CIT supplementation in malnourished elderly women induces a significant decrease in total body fat, as well as a significant increase in appendicular and total lean masses, in comparison with those having a supplementation of non-essential amino acids [13]. Moreover, Figueroa et al. (2015) concluded that after an eight-week intervention, the group EX + CIT had more significant improvements in leg muscle mass (+6% ± 2.2 *p* < 0.005) than the exercise group alone [18]. To the best of our knowledge, this study is the only one that compared a combined intervention in CIT and exercise on a vibration platform, but that was in a population of post-menopausal obese women. Finally, a recent study performed among "Master athletes" tennis women (mean age: 51 years) concluded that CIT supplementation (8 g CIT Malate) one hour before a performance significantly improved grip strength (*p* = 0.042) and muscle power (*p* < 0.001) compared to placebo [56]. However, unlike this study, the observed effect on relative grip strength between groups in our research was due to a difference in the amount of protein and not influenced by an acute dose of CIT supplementation on handgrip strength and muscle power.

Thus, our results show that the CIT–PROT− group significantly decreased its gynoid fat mass and significantly improved its lean body mass and relative grip strength, which seem to confirm these previous studies [13,18]. More specifically, they also confirm the hypothesis of Papadia et al. in which people who are malnourished, or have low protein intake, should benefit from citrulline supplementation to improve muscle function [11]. Citrulline is converted into arginine in the body [57], and arginine stimulates growth hormone secretion [58], which promotes protein synthesis and lipolysis [59].

The main strength of this study is its innovative character, because it is the first time that the influence of the initial amount of protein intake on the combined effect of HIIT and CIT has been investigated among obese older adults. Nevertheless, some limitations must be addressed. Firstly, due to the selection criteria of the participants, the current results are limited to obese older adults. Therefore, the external validity is limited, and future studies should be conducted in other populations, such as among frail or sarcopenic subjects (i.e., people most likely to have functional limitations). Secondly, our results are limited by design, since this study is a posteriori analysis from a larger clinical trial and presents a relatively small sample size. Due to the number of participants, this study should be considered as a preliminary study in this area. However, it should also be considered innovative, because it is also the first to address these issues. Thirdly, the absence of a control group (i.e., with citrulline alone) is another limitation of this work. Indeed, this does not allow us to confirm the potential synergistic effect of HIIT+CIT and initial protein intake. Fourth, although it is a validated and often used method, the three-day food record is not extremely accurate, and may have induced an information bias. Another information bias is that the exercise habits of the participants have not been exhaustively assessed. Since we observed a significant difference in BMI and energy intake between groups, these could be explained, at least partly by the exercise habits. Finally, the intake of proteins was not an intervention and was not controlled either; instead, it was studied a posteriori. The quality of protein ingested (i.e., animal versus vegetal) was also not taken into account. Thus, randomized controlled studies are needed in order to extend the present results. Indeed, this study offers interesting perspectives suggesting the possible role of daily protein intake on the benefits of HIIT when combined with CIT to counteract the loss of muscle strength in the elderly.

## 5. Conclusions

In conclusion, HIIT combined with CITappears to be a very good non-pharmacological avenue for older and obese people to improve their physical health, especially in people consuming low protein. Indeed, our results show that HIIT when combined with CIT induced more and superior positive changes in both groups, compared to exercise alone, independently of the initial amount of protein intake. However, the combination is more beneficial in obese older adults eating initially less than 1 g/kg/day of protein, since they had greater improvements in body composition (i.e., gynoid fat mass and leg fat-free mass) and muscle strength (i.e., relative handgrip strength). These findings support the interest of the combined intervention (exercise + citrulline) and the importance for clinicians to evaluate the protein dietary habits of their older patients. Large randomized controlled trials are needed to extend these promising results and understand the underlying mechanisms responsible for the beneficial action of HIIT + CIT.

## Figures and Tables

**Table 1:** Baseline characteristics of the population (*n* = 73).

Variables	CIT–PROT− (*n* = 19)	PLA–PROT− (*n* = 14)	CIT–PROT+ (*n* = 21)	PLA–PROT+ (*n* = 19)	*p*-Value
Sex (men)	7 (36.8%)	9 (64.3%)	8 (38.1%)	9 (36.8%)	0.46
Age (years)	67.5 ± 4.5	67.8 ± 3.9	66.5 ± 5.2	68.2 ± 3.5	0.65
Weight (kg)	82.0 ± 10.6	81.9 ± 10.6 ^c,d^	76.8 ± 15.0 ^b^	74.9 ± 13.2 ^b^	0.01
Height (cm)	165.6 ± 8.1	168.1 ± 9.5	166.3 ± 7.3	164.4 ± 9.1	0.47
BMI (kg/m²)	30.4 ± 4.0	31.9 ± 6.0 ^d^	27.7 ± 5.0	27.6 ± 3.8 ^b^	0.009
Waist circumference (cm)	104.8 ± 13.6	109.2 ± 10.8	100.6 ± 13.2	101.0 ± 11.9	0.18
Number of steps/d (n)	5591 ± 3068	5288 ±2180	6835 ± 3051	7570 ± 3267	0.10
Energy expenditure (kcal/d)	2115 ± 196	2373 ± 528	2169 ± 364	2183 ± 344	0.26
Energy intake (kcal/d)	1692 ± 418	1754 ± 395	2149 ± 523 ^a,b^	2390 ± 301	0.004
Protein intake (g/kg/d)	0.76 ± 0.17 ^c,d^	0.78 ± 0.15 ^c,d^	1.32 ± 0.23 ^a,b^	1.34 ± 0.32 ^a,b^	<0.001

One-way analysis of variance (ANOVA) was used to compare the four groups. Post-hoc group comparison was performed using Bonferonni correction. Results are presented as mean ± SD. ^a^ Significantly different from CIT–PROT−; ^b^ significantly different from PLA–PROT−; ^c^ significantly different from CIT–PROT+; ^d^ significantly different from PLA–PROT+ (post hoc Bonferroni). BMI: Body Mass Index; PROT−: initial protein intake <1 g/kg/day; PROT+: initial protein intake ≥1 g/kg/day. CIT: supplementation of L-citrulline; PLA: placebo group.

**Table 2:** Effect of initial protein intake on functional capacities and physical endurance.

Variables	Pre-Intervention (T0)				Post-Intervention (T12)				Time Effect	Time*Group Effect
	CIT–PROT−	PLA–PROT−	CIT–PROT+	PROT+PLA	CIT–PROT−	PLA–PROT−	CIT–PROT+	PLA–PROT+		
Functional capacities										
TUG (s)	10.2 ± 1.8	10.7 ± 2.1	9.9 ± 1.4	9.8 ±0.8	8.8 ±1.0	9.4 ± 1.7	8.8 ±1.1	9.0 ± 0.8	<0.001	0.17
TUGf (s)	7.4 ± 1.1	7.6 ± 1.4	7.4 ± 1.1	7.3 ± 0.6	6.4 ± 0.8	6.7 ± 1.2	6.4 ± 1.1	6.6 ± 0.7	<0.001	0.49
Chair stand test (s)	19.5 ± 2.9	20.3 ± 6.0	18.8 ± 3.4	19.5 ± 3.2	16.2 ± 2.8	16.3 ± 4.3	15.4 ± 2.6	16.1 ± 3.7	<0.001	0.89
Alternate step test (n)	28.9 ± 5.0	28.2 ± 4.4	30.4 ± 4.6	28.3 ± 3.7	32.9 ± 5.8	32.4 ± 5.5	34.0 ± 5.2	33.5 ± 4.4	<0.001	0.49
Unipodal balance test (s)	18.1 ± 14.4	24.1 ± 15.4	35.5 ± 18.7	26.2 ± 20.5	30.9 ± 21.9	39.2 ± 20.8	42.6 ± 20.8	35.1 ± 20.4	<0.001	0.65
Physical endurance										
6MWT (m)	547 ± 103	528 ± 83	545 ± 82	566 ± 77	605 ± 89	591 ± 83	626 ± 95	638 ± 86	<0.001	0.70

Data are presented as means ± SD. Time and time*group effects were assessed using ANOVA repeated-measure. *p* < 0.05: significant. TUG = Timed Up and Go. TUGf = fast speed Timed Up and Go. 6MWT = 6-Minute Walking Test.

**Table 3:** Effect of initial protein intake on muscle strength and power.

Variables	Pre-Intervention (T0)				Post-Intervention (T12)				Time Effect	Time*Group Effect
	CIT–PROT−	PLA–PROT−	CIT–PROT+	PLA–PROT+	CIT–PROT−	PLA–PROT−	CIT–PROT+	PLA–PROT+		
Upper and Lower Muscle Strength										
HSr (kg/kg)	0.41 ± 0.12 ^a^	0.41 ± 0.11	0.42 ± 0.11	0.40 ± 0.09	0.45 ± 0.11 ^a^	0.43 ± 0.12	0.43 ± 0.11	0.41 ± 0.08	<0.001	0.04
KESr (N/kg)	9.9 ± 2.2	9.8 ± 2.0	9.9 ± 2.5 ^b^	10.2 ± 1.6	10.3 ± 2.0	10.1 ± 1.9	11.4 ± 1.9 ^b^	10.4 ± 1.6	<0.001	0.07
Muscle Power										
LLMP(W/kg)	8.8 ± 2.4	8.8 ± 3.2	9.1 ± 2.7	9.0 ± 2.1	10.3 ± 2.4	10.7 ± 2.8	10.9 ± 2.9	10.4 ± 1.8	<0.001	0.91

Data are presented as means ± SD. *p* < 0.05: significant. Time and time*group effects were assessed using ANOVA repeated-measure. ^a^ significantly different between T0 and T12, in the PROT− CIT group; ^b^ Significantly different between T0 and T12 in the PROT+ CIT group. HSr = relative to body weight handgrip strength. KESr = relative to body weight knee extensor strength. LLMP = lower limb muscle power.

**Table 4:** Effect of initial protein intake on body composition.

Variables	Pre-intervention (T0)				Post-intervention (T12)				Time Effect	Time*Group Effect
	CIT–PROT−	PLA–PROT−	CIT–PROT+	PLA–PROT+	CIT–PROT−	PLA–PROT−	CIT–PROT+	PLA–PROT+		
Body composition										
Total FM (%)	38.4 ± 7.3 ^a^	37.6 ± 7.8	35.8 ± 5.9 ^b^	38.6 ± 7.7	37.1 ± 7.2 ^a^	37.0 ± 7.2	34.9 ± 5.3 ^b^	38.2 ± 8.1	0.004	0.63
Android (%)	47.4 ± 6.6	47.5 ± 6.0	45.0 ± 9.7	48.0 ± 7.7	46.6 ± 6.7	47.0 ± 5.7	42.9 ± 9.1	47.8 ± 8.4	0.011	0.18
Gynoid (%)	41.2 ± 9.9 ^a^	37.7 ± 11.0	38.6± 7.0	42.2 ± 9.3	39.0 ± 9.0 ^a^	37.7 ± 10.3	38.9 ± 7.47	41.5 ± 10.1	0.07	0.06
LM (kg)	47.5 ± 7.0	51.8 ± 7.3	47.8 ± 7.5	43.1 ± 9.3	48.0 ± 7.2	53.0 ± 7.9	46.3 ± 8.0	43.4 ± 9.5	0.001	0.33
LLM (kg)	16.4 ± 2.4 ^a^	18.4 ± 3.0	16.6 ± 2.9	15.4 ± 3.5	16.9 ± 2.3 ^a^	18.8 ± 3.3	16.6 ± 2.9	15.7 ± 3.5	0.001	0.11
App LM (kg/m)²	21.7 ± 3.67	24.4 ± 4.4	21.7 ± 4.1	20.4 ± 5.2	22.1 ± 3.6	24.7 ± 4.9	21.7 ± 4.2	20.6 ± 5.2	0.02	0.63
Muscle composition										
Thigh muscle Area (cm^2^)	109.9 ± 21.2	91.8 ± 11.9	91.7 ± 28.7	99.3 ± 21.7	109.6 ± 30.7	94.4 ± 15.6	91.3 ± 27.9	95.7 ± 21.8	0.10	0.42
Total Fat Area (cm^2^)	91.9 ± 60.4	93.1 ± 41.6	77.3 ± 38.3	73.1 ± 35.7	90.9 ± 56.1	86.2 ± 37.9	75.4 ± 35.6	66.9 ± 29.9	0.28	0.55
Subcutaneous fat area (cm^2^)	85.7 ± 60.3	87.7 ± 41.5	73.2 ± 38.6	77.7 ± 43.0	85.4 ± 55.0	81.9 ± 38.2	71.3 ± 35.2	62.9± 30.4	0.31	0.59
Intra-muscular fat area (cm^2^)	6.23 ± 1.63	5.35 ± 3.03	4.11 ± 1.93	4.89 ± 2.38	5.46 ± 2.88	4.26 ± 1.92	4.13 ± 1.83	4.05 ± 2.36	0.08	0.67

Data are presented as means ± SD. *p* < 0.05: significant. Time and time*group effects were assessed using ANOVA repeated-measure. ^a^ significantly different between T0 and T12, in the group PROT−CIT; ^b^ significantly different between T0 and T12, in the group PROT+CIT. Total FM = total fat mass. LM = total lean mass. LLM = leg lean mass. App LM = appendicular lean mass.

**Table 5:** Evolution of the parameters, according to the supplementation.

Variables	Δ CIT–PROT−	Δ PLA–PROT−	*p*	Δ CIT–PROT+	Δ PLA–PROT+	*p*
Functional capacities						
TUG (%)	−14.7 ± 7.2	−11.3 ± 9.2	0.30	−10.7 ± 9.4	−7.8 ± 9.1	0.41
TUGf (%)	−13.3 ± 7.2	−11.9 ± 7.2	0.48	−13.8 ± 9.8	−9.6 ± 9.6	0.25
Chair stand test (%)	−16.4 ± 9.0	−18.8 ± 10.2	0.93	−16.6 ± 14.2	−17.2 ± 11.7	0.77
Alternate step test (%)	14.1 ± 9.8	14.9 ± 10.6	0.90	13.2 ± 12.3	18.5 ± 8.9	0.16
Unipodal balance test (%)	124.9± 181.7	77.4 ± 77.2	0.06	51.8 ± 125.7	92.9 ± 141.2	0.79
Physical endurance						
6MWT (%)	12.0 ± 12.6	12.6 (±11.6)	0.71	15.2 ± 9.5	13.6 ± 14.5	0.31
Muscle strength						
HSr (%)	12.4 ± 11.4	3.4 ± 14.6	0.051	3.0 ± 9.8	2.9 ± 9.3	0.92
KESr (%)	6.4 ± 19.1	3.4 ± 8.6	0.74	19.4 ± 23.0	3.6 ± 16.5	0.04
Muscle power						
LLMP (%)	22.7 ± 33.0	25.7 ± 17.4	0.76	24.6 ± 33.9	21.5 ± 33.1	0.59
Body composition						
FM (%)	−3.4 ± 4.1	−1.4 ± 5.4	0.42	−1.8 ± 9.7	−1.1 ± 5.0	0.42
Android (%)	−1.34 ± 4.83	−0.87± 6.53	0.30	−4.04 ± 4.87	−0.74 ± 4.41	0.05
Gynoid (%)	−4.9 ± 5.9	0.4 ± 6.9	0.04	0.9 ± 9.2	−2.1 ± 7.3	0.17
LM (%)	1.3 ± 3.4	2.3 ± 3.8	0.32	0.9 ± 2.4	0.7 ± 3.0	0.73
LLM (%)	3.1 ± 5.2	2.0 ± 3.1	0.30	−0.1 ± 3.6	1.8 ± 5.1	0.24
App LM (%)	1.62 ± 4.71	0.88 ± 3.27	0.39	0.69 ± 2.37	1.24 ± 4.83	0.08
Lean muscle area (%)	−1.76 ± 51.3	2.35 ± 75.2	0.88	−1.73 ± 61.3	127.2 ± 365.9	0.41
Total Fat Area (%)	−6.94 ± 7.28	3.04 ± 23.9	0.06	0.76 ± 22.3	−1.77 ± 9.97	0.81
Total subcutaneous fat area (%)	−5.85 ± 6.54	5.71 ± 28.9	0.13	2.20 ± 21.9	−1.59 ± 9.18	0.61
Intra muscular (%)	1.97 ± 9.56	−0.47 ± 13.9	0.46	−4.69 ± 18.1	1.73 ± 6.33	0.34

Data are presented as means ± SD. Mann–Whitney U-test was used. Δ = % of change between T12 and T0 (T12–T0/T0)*100)). TUG = Timed Up and Go. TUGf = fast speed Timed Up and Go. 6MWT = 6-Minute Walking Test. HSr = relative to body weight handgrip strength. KESr = relative to body weight knee extensor strength. LLMP = Lower limb muscle power. FM = total fat mass. LM = total lean mass. LLM = leg lean mass. App LM = appendicular lean mass.

**Table 6:** Evolution of the parameters, according to the initial amount of protein intake.

Variables	Δ CIT–PROT−	Δ CIT–PROT+	*p*	Δ PLA–PROT−	Δ PLA–PROT+	*p*
Functional capacities						
TUG (%)	−14.7 ± 7.2	−10.7 ± 9.4	0.20	−11.3 ± 9.2	−7.8 ± 9.1	0.48
TUGf (%)	−13.3 ± 7.2	−13.8 ± 9.8	0.88	−11.9 ± 7.2	−9.6 ± 9.6	0.76
Chair stand test (%)	−16.4 ± 9.0	−16.6 ± 14.2	0.73	−18.8 ± 10.2	−17.2 ± 11.7	0.82
Alternate step test (%)	14.1 ± 9.8	13.2 ± 12.3	0.90	14.9 ± 10.6	18.5 ± 8.9	0.28
Unipodal balance test (%)	120 ± 184	52 ± 126	0.16	77 ± 77	101 ± 145	0.88
Physical endurance						
6MWT (%)	12.0 (±12.6)	15.2 (±9.5)	0.27	12.6 (±11.6)	13.6 (±14.5)	0.65
Muscle strength						
HSr (%)	12.4 ± 11.4	3.0 ± 9.8	0.02	3.4 ± 1.6	2.9 ± 9.3	0.60
KESr (%)	6.4 ± 19.1	19.4 ± 23.0	0.06	3.4 ± 8.6	3.6 ± 16.5	0.85
Muscle power						
LLMP (%)	22.7 ± 33.0	24.6 ± 33.9	0.92	25.7 ± 17.4	21.5 ± 33.1	0.32
Body composition						
FM (%)	−3.4 ± 4.1	−1.8 ± 9.7	0.61	−1.4 ± 5.4	−1.1 ± 5.0	0.55
Android (%)	−1.34 ± 4.83	−4.04 ± 8.75	0.05	−0.87 ± 6.53	−0.74 ± 4.41	0.22
Gynoid (%)	−4.9 ± 5.9	0.9 ± 9.2	0.02	0.4 ± 6.9	−2.1 ± 7.3	0.73
LM (%)	1.3 ± 3.4	0.9 ± 2.4	0.75	2.3 ± 3.8	0.7 ± 3.0	0.38
LLM (%)	3.1 ± 5.2	−0.1 ± 3.6	0.02	2.0 ± 3.1	1.8 ± 5.1	0.19
App LM (%)	0.88 ± 3.27	1.24 ± 4.83	0.05	1.62 ± 4.71	0.67 ± 2.37	0.35
Lean Muscle Area (%)	−1.76 ± 51.3	−1.73 ± 61.3	0.69	2.35 ± 75.2	172.2 ± 635.9	0.54
Total Fat Area (%)	−6.94 ± 7.28	0.76 ± 22.3	0.21	3.04 ± 23.9	−1.77 ± 9.97	0.92
Total subcutaneous fat area (%)	−5.85 ± 6.54	2.20 ± 21.9	0.12	5.71 ± 28.9	−1.59 ± 9.18	0.72
Intramuscular (cm²)	1.97 ± 9.56	−4.69 ± 18.1	0.34	−0.47 ± 13.9	1.73 ± 6.33	0.44

Data are presented as means ± SD. Mann–Whitney U-test was used. Δ = % of change between T12 et T0 (T12–T0/T0)*100)). TUG = Timed Up and Go. TUGf = fast speed Timed Up and Go. 6MWT = 6-Minute Walking Test. HSr = relative to body weight handgrip strength. KESr = relative to body weight knee extensor strength. LLMP = Lower limb muscle power. FM = total fat mass. LM = total lean mass. LLM = leg lean mass. App LM = appendicular lean mass.
